# Effect of Comorbidity On Unplanned Readmissions After Percutaneous Coronary Intervention (From The Nationwide Readmission Database)

**DOI:** 10.1038/s41598-018-29303-y

**Published:** 2018-07-24

**Authors:** Chun Shing Kwok, Sara C. Martinez, Samir Pancholy, Waqar Ahmed, Khaled al-Shaibi, Jessica Potts, Mohamed Mohamed, Evangelos Kontopantelis, Nick Curzen, Mamas A. Mamas

**Affiliations:** 1 Keele Cardiovascular Research Group, Centre for Prognosis Research, Keele University, Stoke-on-Trent, UK; 2 Department of Cardiology, Royal Stoke University Hospital, Stoke-on-Trent, UK; 3 Division of Cardiology, Providence St. Peter Hospital, Olympia, Washington USA; 4 The Wright Center for Graduate Medical Education, The Commonwealth Medical College, Scranton, PA USA; 5 Department of Cardiology, King Fahd Armed Forces Hospital, Jeddah, Saudi Arabia; 6 University of Manchester, Manchester, UK; 7 University Hospital Southampton NHS Foundation Trust, Southampton, UK; 8 Faculty of Medicine, University of Southampton, Southampton, UK

## Abstract

It is unclear how comorbidity influences rates and causes of unplanned readmissions following percutaneous coronary intervention (PCI). We analyzed patients in the Nationwide Readmission Database who were admitted to hospital between 2010 and 2014. The comorbidity burden as defined by the Charlson Comorbidity Index (CCI). Primary outcomes were 30-day readmission rates and causes of readmission according to comorbidity burden. A total of 2,294,346 PCI procedures were included the analysis. The patients in CCI = 0, 1, 2 and ≥3 were 842,272(36.7%), 701,476(30.6%), 347,537(15.1%) and 403,061(17.6%), respectively. 219,227(9.6%) had an unplanned readmission within 30 days and rates by CCI group were 6.6%, 8.6%, 11.4% and 15.9% for CCI groups 0, 1, 2 and ≥3, respectively. The CCI score was also associated with greater cost (cost of index PCI for not readmitted vs readmitted was CCI = 0 $21,257 vs $19,764 and CCI ≥ 3 $26,736 vs $27,723). Compared to patients with CCI = 0, greater CCI score was associated with greater independent odds of readmission (CCI = 1 OR 1.25(1.22–1.28), p < 0.001, CCI ≥ 3 OR 2.08(2.03–2.14), p < 0.001). Rates of non-cardiac causes for readmissions increased with increasing CCI group from 49.4% in CCI = 0 to 57.1% in CCI ≥ 3. Rates of early unplanned readmission increase with greater comorbidity burden and non-cardiac readmissions are higher among more comorbid patients.

## Introduction

The average age of patients who undergo percutaneous coronary intervention (PCI) in western populations has increased over time, reflecting an increasingly elderly population who often have comorbid conditions in addition to their prevalent cardiovascular disease^1,2^. In a general population, it is reported that 42% of patients have one or more comorbidties and 23% were multimorbid with the prevalence of multimorbidity increased substantially with age^3^. At least 75% of patients undergoing PCI have at least 1 comorbid condition^4^. While technical advances, improvements in stent platforms, and pharmacology have enabled higher-risk elderly and comorbid patients to be treated with PCI^5^, comorbidity is increasingly recognized as an important prognostic factor in PCI^4,6^. Among patients who undergo PCI, comorbid conditions are typically considered as prevalent individual risk factors for cardiovascular disease, rather than a more holistic measure of comorbid burden where both cardiovascular and non-cardiovascular comorbidities may influence outcomes synergistically rather than in isolation.

The most commonly used measure of comorbid burden is the Charlson comorbidity index (CCI)^4^, derived from several comorbid conditions, that utilizes both the number and the impact of individual comorbidities in order to determine the prognosis of patients with a variety of medical conditions^7^. As a tool, it has been used to estimate prognosis in patients with multiple coexisting illness with a broad range of cardiovascular diseases^6^. CCI has been shown to be an important independent predictor of adverse outcomes following PCI, with previous studies reporting an association between CCI and cardiac death, major adverse cardiovascular events, major bleeding, and stent thrombosis in the setting of PCI^2,4,8^.

Readmissions following PCI are an area of growing interest and contributory mechanisms are not well understood^9,10^. To patients, readmission represents a burden emotionally and represents an adverse clinical outcome. Further, readmissions place a resource burden on healthcare provision. As such, readmissions serve as a surrogate for quality of care which can incur financial penalties for hospitals. Whilst comorbidity burden (as measured by CCI) has been shown to be predictive of unplanned readmission following emergency general surgery^11^, orthopaedic surgery^12^ and in general hospital readmissions^13^, previous work that has studied unplanned readmissions post-PCI has not considered whether there is an association between comorbid burden and the rates or causes of such readmissions. The objective of the current study is to evaluate the comorbidity burden as defined by the CCI and its impact on the rates and causes of 30-day unplanned readmissions after PCI in an unselected national cohort.

## Results

**Figure 1:**
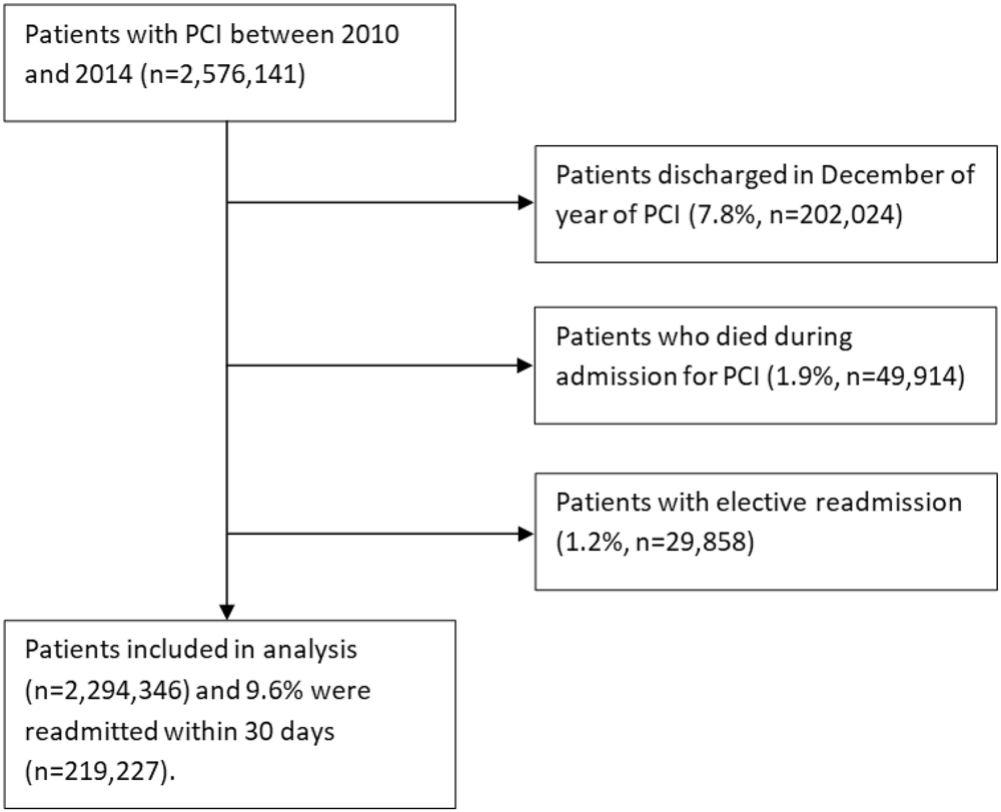
Flow diagram of patients.

**Figure 2:**
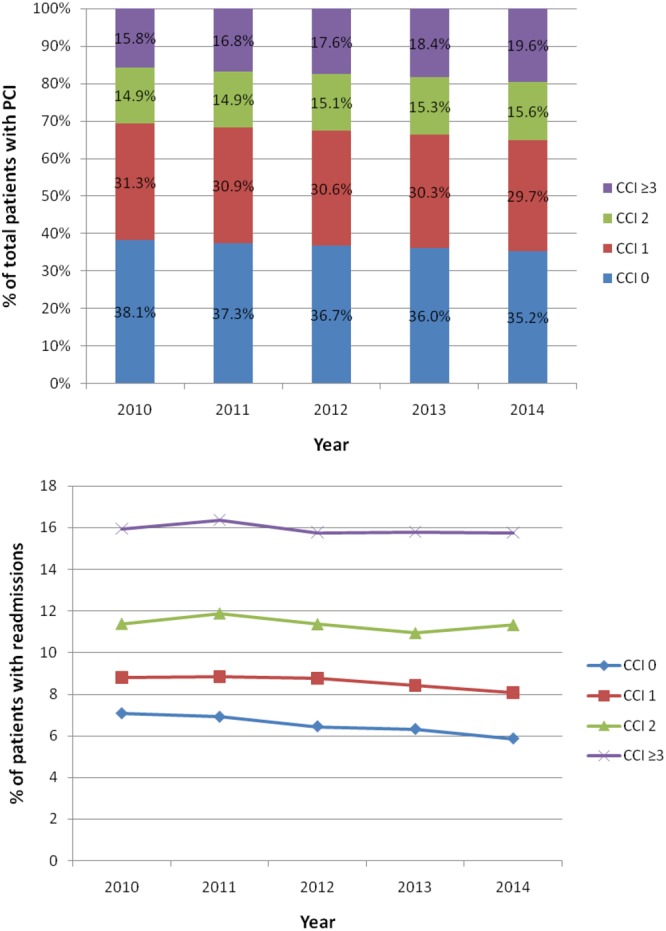
Trends in comorbidity and readmission in patients with percutaneous coronary intervention.

A total of 2,294,346 procedural episodes in patients who survived index PCI were included in the analysis (Fig. 1). The prevalence of comorbidity in patients who undergo PCI has increased over time (Fig. 2). The proportion of patients with a CCI score of ≥3 increased from 15.8% in 2010 to 19.6% in 2014 while the proportion of patients with CCI score of 0 declined from 38.1% in 2010 to 35.2% in 2014. The number of patients with 30-day unplanned readmissions between 2010 and 2014 was 219,227 (9.6%). In CCI groups 0, 1, 2 and ≥3, there were a total of 842,272, 701,476, 347,537 and 403,061 patients, respectively and the rates of unplanned readmissions were 6.6%, 8.6%, 11.4% and 15.9% for the groups CCI = 0, 1, 2 and ≥3.

The trends in readmissions rates of patients according to comorbidity group are shown in Fig. 2. No major changes in readmissions rates were observed over time and the readmission rate for CCI ≥ 3 ranged between 15.8% and 16.4%. The corresponding range for CCI = 0 declined over time from 7.1% to 5.9%.

The characteristics according to CCI group and readmission status is shown in Table 1. In all CCI groups, patients who were older (CCI = 0, 65 vs 62 years; CCI ≥ 3, 68 vs 67 years) and female (CCI = 0, 36.1% vs 27.1%; CCI ≥ 3 40.5% vs 36.3%) were significantly more likely to be readmitted. Patients who underwent an elective PCI procedure were less likely to be readmitted (11.5% vs 16.0% for CCI = 0 and 10.6% vs 16.2% for CCI ≥ 3). Readmitted patients were more likely to be on Medicare (CCI = 0 50.5% vs 40.2%, CCI ≥ 3 77.2% vs 73.0%), and there were fewer patients with private healthcare (CCI = 0 29.5% vs 40.7%, CCI ≥ 3 10.9% vs 15.2%). Alcohol misuse was not significantly different in those readmitted and not readmitted for patients with CCI ≥ 3 (2.5% vs 2.4%, p = 0.63) but significantly different for all other CCI categories. The vast majority of patients were discharged home, but the rates of patients discharged to care homes increased with increasing CCI group. CCI score was also associated with greater cost as the hospital cost of index PCI for CCI = 0, 1, 2 and ≥ 3 for the readmitted compared to not readmitted group was $21,257 vs $19,764, $21,933 vs $20,558, $23,580 vs $22,741 and $26,736 vs $27,723, respectively. Length of stay on index admission was greater for patients who were readmitted in CCI groups 0, 1 and 2 (3.5 vs 2.8 days, 3.9 vs 3.2 days and 4.6 vs 4.1 days, respectively.

**Table 1:** Patient characteristics according to readmission status.

Variable	CCI = 0 (n = 842,272)			CCI = 1 (n = 701,476)			CCI = 2 (n = 347,537)			CCI ≥ 3 (n = 403,061)		
	Not readmitted	Readmitted	p-value	Not readmitted	Readmitted	p-value	Not readmitted	Readmitted	p-value	Not readmitted	Readmitted	p-value
Age	62 ± 13	65 ± 14	<0.001	64 ± 12	66 ± 13	<0.001	67 ± 12	68 ± 13	<0.001	66.8 ± 12	68 ± 13	<0.001
Female	27.1%	36.1%	<0.001	33.3%	41.4%	<0.001	35.6%	42.0%	<0.001	36.3%	40.5%	<0.001
Year			<0.001			<0.001			0.010			0.15
2010	22.6%	24.5%		22.3%	22.9%		21.6%	21.6%		19.7%	19.7%	
2011	20.7%	21.9%		20.6%	21.3%		20.1%	21.1%		19.5%	20.1%	
2012	19.1%	18.8%		19.1%	19.5%		19.0%	19.0%		19.1%	18.9%	
2013	19.6%	18.8%		19.7%	19.3%		20.2%	19.3%		20.9%	20.7%	
2014	18.0%	16.0%		18.2%	17.0%		19.1%	19.0%		20.8%	20.5%	
Elective admission	16.0%	11.5%	<0.001	18.0%	11.8%	<0.001	18.0%	11.5%	<0.001	16.2%	10.6%	<0.001
Weekend admission	21.3%	22.0%	0.007	19.6%	21.3%	<0.001	19.7%	21.5%	<0.001	20.1%	21.7%	<0.001
Diagnosis of AMI	60.1%	58.4%	<0.001	48.8%	50.3%	<0.001	43.7%	45.9%	<0.001	43.4%	47.1%	<0.001
Primary expected payer			<0.001			<0.001			<0.001			<0.001
Medicare	40.2%	50.5%		49.7%	57.6%		61.9%	68.6%		73.0%	77.2%	
Medicaid	6.1%	8.4%		8.3%	10.5%		8.2%	9.7%		7.1%	8.1%	
Private	40.7%	29.5%		31.2%	22.1%		22.1%	15.4%		15.2%	10.9%	
Uninsured	7.7%	6.4%		6.1%	5.3%		3.9%	3.1%		2.0%	1.6%	
No charge	1.0%	1.1%		0.9%	0.9%		0.6%	0.4%		0.3%	0.2%	
Other	4.2%	4.0%		3.9%	3.6%		3.3%	2.7%		2.5%	2.0%	
Median household income (percentile)			<0.001			<0.001			0.001			0.013
0-25^th^	25.7%	27.0%		29.5%	31.4%		31.4%	32.4%		31.5%	32.4%	
26-50^th^	24.7%	24.6%		25.6%	25.7%		25.8%	26.2%		25.3%	25.3%	
51-75^th^	24.9%	24.5%		23.9%	23.4%		23.0%	22.7%		23.1%	22.9%	
76-100^th^	24.7%	23.9%		21.0%	19.5%		19.9%	18.7%		20.1%	19.4%	
Smoking	41.2%	37.5%	<0.001	41.9%	40.1%	<0.001	44.4%	42.4%	<0.001	39.2%	38.1%	0.001
Alcohol misuse	2.8%	3.1%	0.003	2.5%	2.7%	0.035	2.6%	3.0%	0.005	2.4%	2.5%	0.63
Dyslipidemia	66.8%	63.4%	<0.001	75.3%	70.6%	<0.001	75.2%	70.3%	<0.001	72.9%	69.0%	<0.001
Hypertension	64.1%	66.9%	<0.001	77.6%	77.5%	0.63	81.3%	81.1%	0.54	85.8%	86.0%	0.51
Bed size			<0.001			<0.001			0.007			0.046
Small	6.2%	5.4%		5.7%	5.1%		5.4%	4.9%		4.9%	4.6%	
Medium	21.1%	20.6%		20.5%	21.0%		20.4%	19.9%		20.3%	20.7%	
Large	72.7%	74.0%		73.8%	73.9%		74.2%	75.2%		74.8%	74.7%	
Location			0.26			0.26			0.054			0.17
Rural	0.2%	0.1%		0.2%	0.2%		0.2%	0.2%		0.3%	0.2%	
Urban	99.8%	99.9%		99.8%	99.8%		99.8%	99.8%		99.8%	99.8%	
Teaching status			<0.001			0.002			<0.001			<0.001
Nonteaching	46.5%	47.7%		45.2%	46.3%		44.5%	46.2%		43.2%	44.9%	
Teaching status	53.5%	52.3%		54.8%	53.7%		55.5%	53.8%		56.8%	55.1%	
Multivessel disease	14.7%	15.1%	0.14	16.4%	15.8%	0.008	17.0%	16.5%	0.099	18.1%	17.7%	0.16
Bifurcation disease	2.8%	2.6%	0.074	2.8%	2.7%	0.38	3.0%	2.7%	0.048	3.1%	3.0%	0.19
Circulatory support	2.7%	4.9%	<0.001	2.7%	4.1%	<0.001	3.0%	3.7%	<0.001	3.7%	3.9%	0.29
Vasopressor use	0.4%	0.6%	<0.001	0.4%	0.5%	<0.001	0.5%	0.6%	0.071%	0.6%	0.7%	0.40
IABP use	2.6%	4.6%	<0.001	2.4%	3.7%	<0.001	2.6%	3.2%	<0.001	3.0%	3.2%	0.081
FFR use	1.7%	1.6%	0.61	1.9%	1.8%	0.34	2.1%	2.1%	0.78	2.2%	2.5%	0.005
IVUS use	6.9%	7.0%	0.52	7.0%	7.0%	0.90	7.2%	6.9%	0.20	7.2%	6.8%	0.016
DES	76.0%	69.6%	<0.001	75.6%	69.7%	<0.001	72.5%	67.4%	<0.001	69.7%	66.0%	<0.001
Complete heart block	0.9%	1.2%	<0.001	0.9%	1.0%	0.33	1.0%	1.2%	0.010	1.3%	1.2%	0.83
Stroke or TIA	1.3%	1.9%	<0.001	2.4%	2.7%	0.006	4.3%	4.3%	0.93	6.7%	6.0%	<0.001
Cardiogenic shock	2.3%	4.0%	<0.001	2.4%	1.7%	<0.001	2.9%	3.8%	<0.001	3.7%	3.9%	0.17
Cardiac arrest	1.9%	2.6%	<0.001	1.4%	1.7%	<0.001	1.6%	1.7%	0.26	2.1%	1.7%	<0.001
Acute kidney injury	0.3%	0.6%	<0.001	0.4%	0.8%	<0.001	0.6%	1.1%	<0.001	1.3%	1.4%	0.097
Bleeding	0.4%	0.7%	<0.001	0.5%	0.9%	<0.001	0.7%	1.1%	<0.001	1.3%	1.5%	0.041
Blood transfusion	0.04%	0.08%	0.002	0.05%	0.10%	<0.001	0.06%	0.07%	0.77	0.05%	0.08%	0.036
Vascular complication	0.6%	0.8%	<0.001	0.7%	1.0%	<0.001	1.0%	1.2%	0.001	1.2%	1.3%	0.035
Emergency CABG	1.2%	1.8%	<0.001	1.4%	1.8%	<0.001	1.6%	1.3%	0.011	1.3%	1.0%	<0.001
Discharge location			<0.001			<0.001			<0.001			<0.001
Home (self-care)	94.2%	87.3%		91.2%	83.2%		85.2%	76.0%		74.3%	66.2%	
Short term hospital	0.4%	0.8%		0.4%	0.6%		0.4%	0.7%		0.7%	0.8%	
Transfer to other institution	1.6%	4.0%		2.7%	5.4%		5.4%	8.3%		10.7%	13.3%	
Care home	3.4%	7.2%		5.3%	9.9%		8.5%	14.3%		13.9%	18.9%	
Discharge against medical advice or discontinue care	0.4%	0.8%		0.4%	0.8%		0.4%	0.8%		0.4%	0.8%	
Length of stay	2.8 ± 3.5	3.5 ± 3.1	<0.001	3.2 ± 4.2	3.9 ± 3.3	<0.001	4.1 ± 5.8	4.6 ± 3.9	<0.001	6.3 ± 8.6	6.0 ± 6.7	<0.001
Cost of index PCI	$19,764 ± 14,066	$21,257 ± 13,460	<0.001	$20,558 ± 15,948	$21,933 ± 13,622	<0.001	$22,741 ± 19,409	$23,580 ± 14,7044	<0.001	$27,723 ± 26,288	$26,736 ± 16,559	<0.001

**Figure 3:**
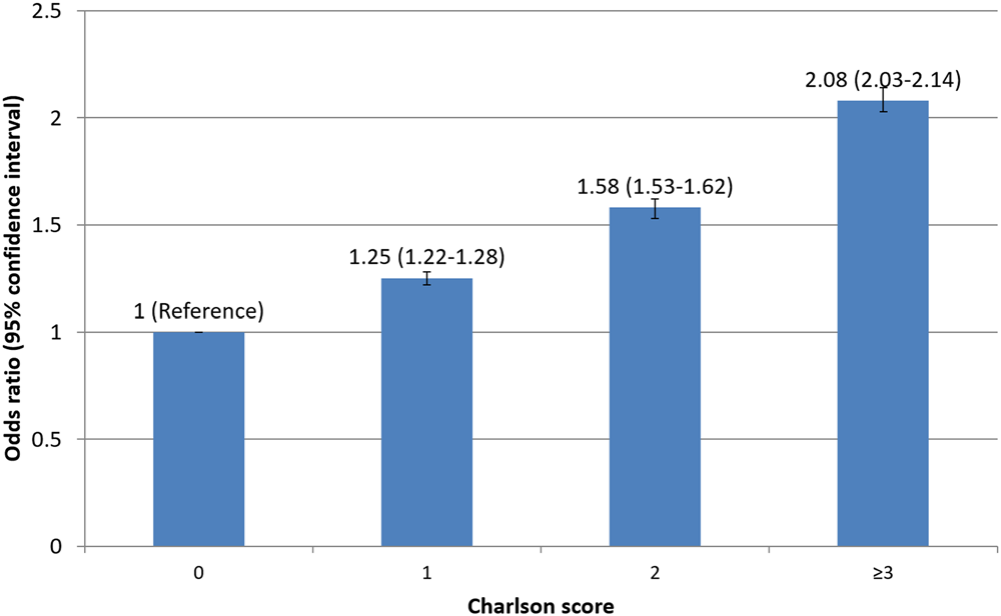
Adjusted odds of readmission by comorbidity.

**Figure 4:**
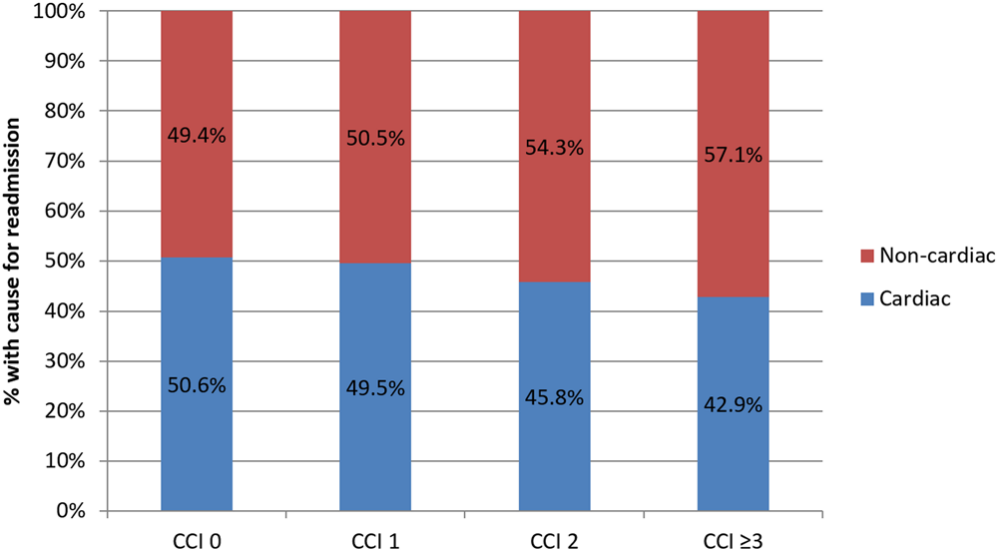
Cause of readmission by comorbidity group.

**Figure 5:**
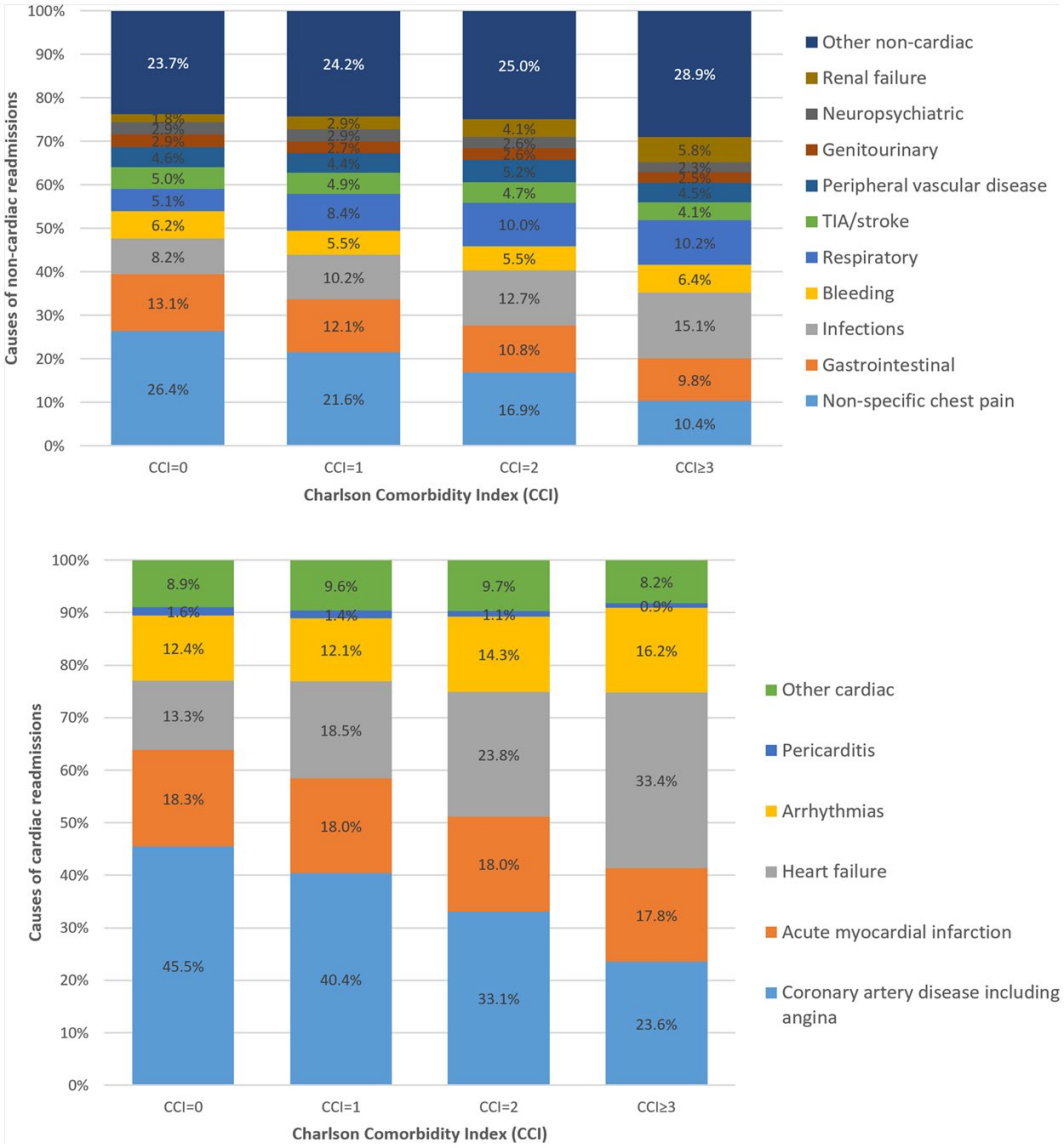
Causes of non-cardiac and cardiac readmissions by comorbidity group.

Following adjustment for baseline covariates not already included in the CCI score, greater CCI score was associated with greater independent odds of readmission (CCI = 1 OR 1.25 95%CI 1.22–1.28, p < 0.001, CCI = 2 OR 1.58 95%CI 1.53–1.62, p < 0.001, CCI ≥ 3 OR 2.08 95%CI 2.03–2.14, p < 0.001) compared to patients with CCI = 0, (Fig. 3). The causes of readmissions by CCI group are shown in Table 2 and Figs 4 and 5. Rates of non-cardiac causes for readmissions increased with increasing CCI group from 49.4% in CCI = 0 to 57.1% in CCI ≥ 3. For non-cardiac causes of readmission, increase in CCI score was associated with a higher prevalence of readmission for respiratory diseases and a lower prevalence of readmission due to non-specific chest pain and gastrointestinal disease. For cardiac causes of readmission, a higher CCI score was associated with a higher prevalence of readmission for heart failure and arrhythmias and a lower prevalence of readmission related to coronary artery disease including angina and acute myocardial infarction. The cause of other non-cardiac readmissions is shown in Supplementary Table 2.

**Table 2:** Cause of readmission by comorbidity group.

Top 10 non-cardiac causes of readmission	CCI 0*	CCI 1*	CCI 2*	CCI 3*
Non-specific chest pain	26.4%	21.6%	16.9%	10.4%
Gastrointestinal	13.1%	12.1%	10.8%	9.8%
Infections	8.2%	10.2%	12.7%	15.1%
Bleeding	6.2%	5.5%	5.5%	6.4%
Respiratory	5.1%	8.4%	10.0%	10.2%
TIA/stroke	5.0%	4.9%	4.7%	4.1%
Peripheral vascular disease	4.6%	4.4%	5.2%	4.5%
Genitourinary	2.9%	2.7%	2.6%	2.5%
Neuropsychiatric	2.9%	2.9%	2.6%	2.3%
Renal failure	1.8%	2.9%	4.1%	5.8%
**Cardiac causes of readmission**	CCI 0**	CCI 1**	CCI 2**	CCI 3**
Coronary artery disease including angina	45.5%	40.4%	33.1%	23.6%
Acute myocardial infarction	18.3%	18.0%	18.0%	17.8%
Heart failure	13.3%	18.5%	23.8%	33.4%
Arrhythmias	12.4%	12.1%	14.3%	16.2%
Pericarditis	1.6%	1.4%	1.1%	0.9%
Other cardiac	8.9%	9.6%	9.7%	8.2%

*% of non-cardiac cause of readmissions.

**% of cardiac cause of readmissions.

The outcomes for readmissions are shown in Table 3. The length of stay for the readmission was higher with greater CCI (CCI = 0 3.5 days, CCI = 1 3.9 days, CCI = 2 4.5 days, CCI ≥ 3 5.2 days, p < 0.001) as was the cost of the readmission (CCI = 0 $10,974, CCI = 1 $11,532, CCI = 2 $12,064, CCI ≥ 3 $13,177, p < 0.001). The mortality rates for the readmission increased with higher CCI score from 1.7% in CCI = 0 to 3.9% CCI ≥ 3 (p < 0.001).

**Table 3:** Outcomes for readmission by comorbidity group.

Readmission	CCI 0 (n = 55,224)	CCI 1 (n = 60,293)	CCI 2 (n = 39,525)	CCI ≥ 3 (n = 64,185)	p-value
Length of stay	3.5 ± 4.6	3.9 ± 5.1	4.5 ± 5.4	5.2 ± 6.1	<0.001
Cost	$10,974 ± 16,617	$11,532 ± 16,049	$12,064 ± 16,574	$13,177 ± 17,807	<0.001
Death	1.7%	2.3%	3.0%	3.9%	<0.001

## Discussion

Comorbidity burden in patients who undergo PCI is associated with different rates of early unplanned readmissions and is associated with prolonged length of stay in hospital, increased hospital cost, and greater mortality during the readmission episode. Our analysis shows that with increasing comorbid burden, there is an increase in non-cardiac causes for readmission. Finally, we show that causes of unplanned readmissions vary according to comorbid burden, with increasing comorbidity burden associated with a greater incidence of readmissions for respiratory causes, heart failure and arrhythmias, whilst patients with no significant comorbid burden were more likely to be readmitted with non-specific chest pain, gastrointestinal disease and manifestations of coronary artery disease such as angina.

Our data show that the comorbid burden of patients undergoing PCI has increased over time, but rates of readmission are consistently higher across all years at the highest comorbidity burden. While previous studies undertaken using national data have examined comorbidities in PCI, these studies have treated each comorbidity as an individual isolated factor rather than using global measures of comorbid burden, and have only considered comorbidities and adverse outcomes for the index PCI hospitalization^14–17^. To the best of our knowledge, this analysis is the first to report the relationship between global comorbid burden (made up of both cardiovascular and non-cardiovascular comorbidities) and unplanned hospital readmissions following PCI. For some patients, the risk of readmission may be greater as a direct consequence of their non-cardiac comorbidities rather than cardiac pathology or complications from the PCI procedure. The reasons for this are unclear. Comorbidities are complex because they can work independently or synergistically to modulate the propensity for patients to have readmissions related to cardiac disease, PCI related complications, and non-cardiac existing illnesses. Patients with comorbidities have an increased potential for adverse drug reactions^18^ and the liabilities of polypharmacy in medication adherence^19^. In the current study, we have shown the CCI score, a measure of global comorbid burden is independently associated with increased cost, length of stay and readmission following PCI. This builds on our previous work which demonstrates that CCI was independently associated with cardiac death, major adverse cardiovascular events, major bleeding, and stent thrombosis in a much smaller multicentre registry of patients undergoing PCI^4^.

We show that differences in causes for readmission depend on the comorbidity burden in patients who undergo PCI. We observe that the prevalence of readmissions for infections and respiratory causes increase with greater comorbidity burden. Whilst it is not understood how chronic comorbid medical conditions influence risk of infections, there is evidence that certain diseases such as diabetes, chronic liver disease and cancer can increase the risk of sepsis^20^. In our current cohort, we found that the prevalence of chronic lung disease was high in the groups with comorbidity (CCI groups 1, 2 and ≥3 rates were 18%, 34% and 36% respectively). Chronic obstructive pulmonary disease is likely to represent a large population of the chronic lung disease in the current study and patients who have chronic obstructive pulmonary disease are known to have high rates of readmissions (10%) within 30 days of COPD related admissions^21^. Prevalence of renal failure as a cause of readmission increased with greater CCI score to 5.6% with CCI ≥ 3.

Our results have several clinical implications. First, the patients with high comorbid burden are a high-risk group for readmissions. This suggests that patients with a high comorbidity burden should be assessed prior to discharge to ensure that their comorbidities are well-managed with appropriate follow up. To date, there has been one multimodal intervention which has been shown to reduce readmissions after PCI from 9.6% to 5.3%^22^. This intervention involves several measures including patient assessment with a validated risk score which contains 3 comorbidities, discharge checklist, patient education video, follow-up clinic, and computerized notification system for attendance to emergency departments within 30 days. The discharge checklist advised instructions for managing heart failure, diabetes and other secondary diagnoses but did not specifically explain what these instructions were or how exactly to manage comorbidities. Secondly, assessment of comorbidity may be useful in predictive scores for readmissions. There are several published scores designed to predict readmission^23,24^, but only one which has been studied in a PCI population^22^. This contained the comorbidities chronic heart failure, chronic lung disease and peripheral artery disease but global comorbid burden was not considered, which maybe more important than these isolated comorbidities.

Our study has several strengths. First, this largest national study of patients who undergo PCI enables sufficient sample size to consider the Charlson score and its relationship with readmissions. Secondly, the Nationwide Readmission Database is 99% complete for the variables in the current study. The data is designed to be generalizable to hospitals in the United States rather than specific to a geographic area. Finally, we were able to consider the effect of a variety of patient characteristics, hospital-related, PCI-related and outcome-related variables and how they influence readmission rates.

Our study also has a few limitations. Firstly, there is no possible linkage between years as the data is derived from five unique datasets corresponding to each year between 2010 to 2014. We excluded patients who were discharged in the month of December in order to ensure adequate 30-day follow up. Another limitation was that the NRD dataset did not have data on completeness of revascularisation or procedural success and the use of discharge medications such as type and duration of dual antiplatelet therapy. Applicability of the data is extrapolated to represent the country’s trends, as the collected data in the current analysis is derived from administrative claims from 21 states where regional heterogeneity could not be explored. In addition, we cannot exclude possible bias from coding errors, and we had to use the primary discharge diagnosis codes for causes of readmissions, which may be subject to bias. On the converse, we cannot know the true health status of all patients, as diagnoses exist when they are discovered. Arnold *et al*. demonstrated the rate of previously undiagnosed diabetes in patients presenting with AMI as 10%, although only 1/3 of those patients are identified, and only 5% of those with undiagnosed diabetes receive treatment at 6 months post discharge^25^. Although diabetes can be diagnosed easily with blood tests, other comorbid conditions such as COPD and occult malignancy may not present to clinical attention until the unplanned readmission. Finally, studies of readmissions may be affected by bias related to survivorship. Patients who died during the index PCI hospitalization are not considered and patients who died out of the hospital are not captured in the present analysis.

In conclusion, our results demonstrate that comorbidity is common in PCI and associated with early unplanned readmissions. The causes of readmissions are influenced by comorbidity where patients with higher comorbidity show a greater prevalence of non-cardiac causes for readmissions. These findings suggest that comorbidity should be considered in risk assessments for patients who undergo PCI and patient with high comorbid burden should be targeted for interventions which can reduce readmissions.

## Methods

The Nationwide Readmissions Database (NRD) contains national hospitalization and rehospitalization data for patients of all ages within the United States, which is produced by the Healthcare Cost and Utilization Project of the Agency for Healthcare Research and Quality^26^. The data is derived from discharge-level data for hospitalizations from 21 geographically-dispersed states and is designed to represent 49.1% of all US hospitalizations^9^. The NRD contains a de-identified unique patient linkage number, which allows for the determination of readmissions by tracking of patients across hospitals within a calendar year.

We included men and women, aged 18 years or older, who underwent PCI with discharge dates between 2010 and 2014 with 30-day follow up. The NRD contains up to 15 procedural codes for each inpatient admission. PCI was defined by the procedural code 066 (PTCA OR CORONARY ATHER), 3606 (INSERT CORON ART STENT) and 3607 (INSERT DRUG ELUTING CRNRY AR)^10^.

The NRD also contains International Statistical Classification of Disease and Related Health Problems (ICD) 9 codes, Elixhauser comorbidity codes and Clinical Classification Software (CCS) codes. The Charlson Comorbidity Index (CCI) is a measure of co-morbidity burden which provides a measure of the prognostic impact of 17 comorbid conditions which considers the number of comorbidities and their individual prognostic value^6^. We used the Elixhauser comorbidity variables and the paralysis variable from Elixhauser comorbidities as a surrogate for hemiplegia and the connective tissue disease and leukemia from the CCS codes 210, 211 & 39 respectively to calculate the CCI. The continuous CCI score was then categorized into comorbidity groups with CCI score of 0, 1, 2 and ≥3^27^. Patients were excluded if they died during index PCI, discharged during the month of December and had a planned readmission within 30 days.

Several variables were collected in the analysis. Age, sex, year, elective admission, weekend admission, primary expected payer, median household income, hospital-bed number, location, teaching status, discharge location, length of stay, and cost were available in the NRD dataset. Diagnosis of acute myocardial infarction was defined as a first diagnostic code of 410*1 or 4111. Using the ICD-9 diagnostic codes we defined several patient variables including smoking status (V1582 3051), dyslipidemia (2720–2724), complete heart block (4260), stroke or transient ischemic attack (431 433*1 434*1 435* 4336* 99701), cardiogenic shock (78551), cardiac arrest (4275), acute kidney injury (4590* 56881 5789* V582* 431* 4329*) and bleeding (4590* 56881 5782* 431* 4329*). Vascular complications were determined by ICD-9 diagnostic codes (900–904 9982 9992 9977 86804) and ICD-9 procedural codes (3931 3941 3949 3952 3956 3957 3959 3979). ICD-9 procedural codes were used to define receipt of circulatory support (3761 3768 3965), multivessel disease (0041 0042 0043 0046 0047 0048), bifurcation disease (0044), vasopressor use (0017), intraaortic balloon pump use (3761), fractional flow reserve (0059), intravascular ultrasound (0024), drug eluting stent (3607) and blood transfusion (9900). Elixhauser comorbidity code was used to define alcohol misuse.

The primary outcome of the study was 30-day unplanned all-cause readmissions and the causes of readmissions. The causes of readmission were defined by the first diagnosis based on the Clinical Classification Software codes which are shown in Supplementary Table 1.

Statistical analysis was performed on Stata 14.0 (College Station, TX). Descriptive statistics are presented according to comorbidity group and readmission status for all included variables. The one-way analysis of variance was used to compare continuous variables while the Chi^2^ test was used to compare categorical variables according to comorbidity group. Trends in the prevalence of patients in each comorbidity group and readmission rates are shown graphically. Simple and multiple logistic regressions adjusted for all variables were used to identify the effect of comorbidity group on 30-day readmissions. Causes of readmission were explored in a table. A flow diagram was used to describe patient outcomes for both admission and readmissions. In addition, for the analysis of the odds of readmission by CCI score we adjusted for the baseline covariates not included in the CCI score which included age, sex, year, elective PCI, weekend procedure, acute myocardial infarction, primary expected payer, income, smoking, alcohol, dyslipidaemia, hypertension, hospital bed size, urban hospital, teaching hospital, multivessel PCI, bifurcation PCI, circulatory support, vasopressor use, intra-aortic balloon pump use, fractional flow reserve, intravascular ultrasound, drug eluting stent, complete heart block, stroke or TIA, cardiac arrest, acute kidney injury, bleeding, vascular complication, emergency CABG and discharge location.

### Data availability statement

The data in the current manuscript is not available.

### Ethical approval and informed consent

The study is an analysis of anonymized data and ethical approval and informed consent was not required.

## Electronic supplementary material

Supplementary Data
