# Supplementary material for: Effect of Comorbidity On Unplanned Readmissions After Percutaneous Coronary Intervention (From The Nationwide Readmission Database)
Source: Sci Rep. 2018 Jul 24;8:11156. doi: 10.1038/s41598-018-29303-y (PMC6057975; doi:10.1038/s41598-018-29303-y)
Supplement: Supplementary file 1 — Supplementary Data [file 41598_2018_29303_MOESM1_ESM.pdf]

Title:

Effect of Comorbidity On Unplanned Readmissions After Percutaneous Coronary Intervention (From The Nationwide Readmission Database)

Author list:

Chun Shing Kwok, Sara C. Martinez, Samir Pancholy, Waqar Ahmed, Khaled al-Shaibi, Jessica Potts, Mohamed Mohamed, Evangelos Kontopantelis, Nick Curzen, Mamas A Mamas

Supplementary Table 1: Classification of CCS codes for Readmissions Causes

Causes of readmission	CCS code	Diagnosis
Respiratory	127	Chronic obstructive pulmonary disease and bronchietasis
	128	Asthma
	130	Pleurisy, pneumothorax, pulmonary collapse
	131	Respiratory failure, insufficiency and arrest
	132	Lung disease due to external agents
	133	Other lower respiratory disease
	134	Other upper respiratory disease
	221	Respiratory distress syndrome
Infection	1	Tuberculosis
	2	Septicemia
	3	Bacterial infection
	4	Mycoses
	5	HIV infection
	6	Hepatitis
	7	Viral infection
	8	Other infection
	9	Sexually transmitted infection
	76	Meningitis
	77	Encephalitis
	78	Other CNS infection and poliomyelitis
	90	Inflammation or infection of eye
	122	Pneumonia
	123	Influenza
	124	Acute and chronic tonsillitis
	125	Acute bronchitis
	126	Other upper respiratory infections
	129	Aspiration pneumonitis
	135	Intestinal infection
	197	Skin and subcutaneous tissue infections
	201	Infective arthritis and osteomyelitis (except that caused by tuberculosis or sexually transmitted disease)
Bleeding	60	Acute posthaemorrhagic anemia
	153	Gastrointestinal hemorrhage
	182	Hemorrhage during pregnancy; abrutio placenta; placenta previa
Peripheral vascular disease	114	Peripheral and visceral atherosclerosis
	115	Aortic, peripheral and visceral artery aneurysms
	116	Aortic and peripheral arterial embolism or thrombosis
	117	Other circulatory disease
	118	Phlebitis, thrombophlebitis and thromboembolism
	119	Varicose veins of lower extremities
Genitourinary	159	Urinary tract infection
	160	Calculus of the urinary tract

	161	Other diseases of kidney and ureters
	162	Other diseases of bladder and urethra
	163	Genitourinary symptoms and ill-defined conditions
	164	Hyperplasia of prostate
	165	Inflammatory conditions of the male genital organs
	166	Other male genital disorders
	170	Prolapse of female genital organs
	175	Other female genital disorders
	215	Genitourinary congenital anomalies
Renal disease	156	Nephritis; nephrosis; renal sclerosis
	157	Acute and unspecified renal failure
	158	Chronic kidney disease
Gastrointestinal	138	Esophageal disorders
	139	Gastroduodenal ulcer (except hemorrhage)
	140	Gastritis and duodenitis
	141	Other disorders of stomach and duodenum
	142	Appendicitis and other appendiceal conditions
	143	Abdominal hernia
	144	Regional enteritis and ulcerative colitis
	145	Intestinal obstruction without hernia
	146	Diverticulosis and diverticulitis
	147	Anal and rectal conditions
	148	Peritonitis and intestinal abscess
	149	Biliary tract disease
	150	Liver disease; alcohol-related
	151	Other liver diseases
	152	Pancreatic disorders (not diabetes)
	154	Noninfectious gastroenteritis
	155	Other gastrointestinal disorders
	214	Digestive congenital anomalies
	222	Hemolytic jaundice and perinatal jaundice
	250	Nausea and vomiting
	251	Abdominal pain
TIA/stroke	109	Acute cerebrovascular disease
	110	Occlusion of stenosis of precerebral arteries
	111	Other and ill-defined cerebrovascular disease
	112	Transient cerebral ischemia
	113	Late effects of cerebrovascular disease
Trauma	207	Pathological fracture
	225	Joint disorders and dislocations; trauma-related
	226	Fracture of neck of femur (hip)
	227	Spinal cord injury
	228	Skull and face fractures
	229	Fracture of upper limb
	230	Fracture of lower limb

	231	Other fractures
	232	Sprains and strains
	233	Intracranial injury
	234	Crushing injury or internal injury
	235	Open wounds of head; neck; and trunk
	236	Open wounds of extremities
	239	Superficial injury; contusion
	244	Other injuries and conditions due to external causes
	260	All (external causes of injury and poisoning)
Endocrine/metabolic	48	Thyroid disorders
	49	Diabetes mellitus without complication
	50	Diabetes mellitus with complication
	51	Other endocrine disorders
	53	Disorders of lipid metabolism
	58	Other nutritional and endocrine/metabolic disorders
	186	Diabetes or abnormal glucose tolerance complicating pregnancy; childbirth; or the puerperium
Neuropsychiatric	650	Adjustment disorders
	651	Anxiety disorders
	652	Attention-deficit, conduct, and disruptive behavior disorders
	653	Delirium, dementia, and amnestic and other cognitive disorders
	654	Developmental disorders
	655	Disorders usually diagnosed in infancy and childhood or adolescence
	656	Impulse control disorders, NEC
	657	Mood disorders
	658	Personality disorders
	659	Schizophrenia and other psychotic disorders
	660	Alcohol-related disorders
	661	Substance-related disorders
	662	Suicide and intentional self-inflicted injury
	663	Screening and history of mental health and substance abuse codes
	670	Miscellaneous mental health disorders
	79	Parkinson's disease
	80	Multiple sclerosis
	81	Other hereditary and degenerative nervous system conditions
	82	Paralysis
	83	Epilepsy, convulsions
	84	Headache including migraine
	85	Coma, stupor and brain damage
	95	Other nervous system disorders
	216	Nervous system congenital anomalies
	650	Adjustment disorders
	651	Anxiety disorders
	652	Attention-deficit, conduct, and disruptive behavior disorders
	653	Delirium, dementia, and amnestic and other cognitive disorders

	654	Developmental disorders
	655	Disorders usually diagnosed in infancy and childhood or adolescence
	656	Impulse control disorders, NEC
	657	Mood disorders
	658	Personality disorders
	659	Schizophrenia and other psychotic disorders
	660	Alcohol-related disorders
	661	Substance-related disorders
	662	Suicide and intentional self-inflicted injury
	663	Screening and history of mental health and substance abuse codes
	670	Miscellaneous mental health disorders
Hematological/neoplastic	11	Cancer of head and neck
	12	Cancer of esophagus
	13	Cancer of stomach
	14	Cancer of colon
	15	Cancer of rectum and anus
	16	Cancer of liver and intrahepatic bile ducts
	17	Cancer of pancreas
	18	Cancer of other GI organs, peritoneum
	19	Cancer of bronchus, lung
	20	Cancer of other respiratory and intrathoracic
	21	Cancer of bone and connective tissue
	22	Melanoma of skin
	23	Other non-epithelial cancer of skin
	24	Cancer of breast
	25	Cancer of uterus
	26	Cancer of cervix
	27	Cancer of ovary
	28	Cancer of other female genital organs
	29	Cancer of prostate
	30	Cancer of testis
	31	Cancer of other male genital organs
	32	Cancer of bladder
	33	Cancer of kidney and renal pelvis
	34	Cancer of other urinary organs
	35	Cancer of brain and nervous system
	36	Cancer of thyroid
	37	Hodgkin's disease
	38	Non-Hodgkin's lymphoma
	39	Leukaemias
	40	Multiple myeloma
	41	Cancer, other and unspecified primary
	42	Secondary malignancies
	43	Malignant neoplasm without specification of site

	44	Neoplasm of unspecified nature or uncertain behaviour
	46	Benign neoplasm of uterus
	47	Other and unspecified benign neoplasm
	59	Deficiency and other anemias
	61	Sickle cell anemia
	62	Coagulation and hemorrhagic disorders
	63	Disease of white blood cells
	64	Other hematologic conditions
Rheumatology problem	54	Gout and other crystal arthropathies
Opthalmology problem	86	Cataract
	87	Retinal detachment defects, vascular occlusion and retinopathy
	88	Glaucoma
	89	Blindness and vision defects
	91	Other eye disorders
ENT problem	92	Otitis media and related conditions
	93	Conditions associate with dizziness or vertigo
	94	Other ear and sense organ disorder
Non-specific chest pain	102	Non-specific chest pain
Oral health problem	136	Disorders of teeth and jaw
	137	Diseases of mouth; excluding dental
Obstetric admission including pregnancy	174	Female infertility
	176	Contraceptive and procreative management
	177	Spontaneous abortion
	178	Induced abortion
	179	Postabortion complication
	180	Ectopic pregnancy
	181	Other complications of pregnancy
	184	Early or threatened labor
	185	Prolonged pregnancy
	187	Malposition; malpresentation
	188	Fetopelvic disproportion; obstruction
	189	Previous C-section
	190	Fetal distress and abnormal forces of labor
	191	Polyhydramnios and other problems of amniotic cavity
	192	Umbilical cord complication
	193	OB-related trauma to perineum and vulva
	194	Forceps delivery
	195	Other complications of birth; puerperium affecting management of mother
	196	Other pregnancy and deliver including normal
	218	Liveborn
	219	Short gestation; low birth weight; and fetal growth retardation
	220	Intrauterine hypoxia and birth asphyxia
	223	Birth trauma

	224	Other perinatal conditions
Dermatology problem	198	Other inflammatory condition of skin
	199	Chronic ulcer of skin
	200	Other skin disorders
Poisoning	241	Poisoning by psychotrophic agents
	242	Poisoning by other medication and drugs
	243	Poisoning by nonmedical substances
Syncope	245	Syncope
Other non-cardiac	10	Immunization and screening for infectious disease
	45	Maintenance chemotherapy, radiotherapy
	52	Nutritional deficiencies
	55	Fluid and electrolyte disorders
	56	Cystic fibrosis
	57	Immunity disorder
	120	Hemorrhoids
	121	Other diseases of veins and lymphatics
	167	Nonmalignant breast conditions
	168	Inflammatory disease of female pelvic organs
	169	Endometriosis
	172	Ovarian cyst
	173	Menopausal disorders
	202	Rheumatoid arthritis and related disease
	203	Osteoarthritis
	204	Other non-traumatic joint disorders
	205	Spondylosis; intervertebral disc disorders; other back problems
	206	Osteoporosis
	208	Acquired foot deformities
	209	Other acquired deformities
	210	Systemic lupus erythematosus and connective tissue disorders
	211	Other connective tissue disease
	212	Other bone disease and musculoskeletal deformities
	217	Other congenital anomalies
	237	Complication of device; implant or graft
	238	Complications of surgical procedure or medical care
	240	Burns
	246	Fever of unknown origin
	247	Lymphadenitis
	248	Gangrene
	252	Malaise and fatigue
	253	Allergic reactions
	254	Rehabilitation care; fitting of prostheses; and adjustment of devices
	255	Administrative/social admission
	256	Medical examination/evaluation
	257	Other aftercare
	258	Other screening for suspected conditions (not mental disorders or infectious

		disease)
	259	Residual codes; unclassified
Heart failure	108	Congestive heart failure non-hypertensive
Arrhythmia	106	Cardiac dysrrhythmias
	107	Cardiac arrest and ventricular fibrillation
Conduction disorder	105	Conduction disorders
Valve disorders	96	Heart valve disorder
Hyper/hypotension	98	Essential hypertension
	99	Hypertension with complications and secondary hypertension
	183	Hypertension complicating pregnancy; childbirth and the puerperium
	249	Shock
Pericarditis	97	Peri-, endo- and myocarditis, cardiomyopathy
Coronary artery disease including angina	101	Coronary atherosclerosis and other heart disease
Acute myocardial infarction	100	Acute myocardial infarction
Others (cardiac)	103	Pulmonary heart disease
	104	Other and ill-defined heart disease
	213	Cardiac and circulatory congenital anomalies

Supplementary Table 2: Causes of other non-cardiac readmissions

**Causes of other non-cardiac readmissions**	**%**
Haematological/neoplasm	11.96
Endocrine/metabolic	10.26
Trauma	9.41
Fluid and electrolyte disorder	7.71
Syncope	7.34
Rheumatological	6.86
Vascular complication	6.61
ENT problem	5.91
Post-procedure haemorrhage/hematoma/seroma	5.26
Post-operative infection, seroma, fistula and non-healing wound	3.26
Infection/inflammation of device implant, graft, catheter	2.85
Hypotension	2.57
Fever, chills and malaise	2.46
Other complication of device implant, graft, catheter	2.17
Poisoning	2.10
Dermatological	1.35
Haemorrhoids and varices	1.33
Atherosclerosis of graft, vein or artery	0.86
Infection due to central venous catheter	0.78
Generalized pain	0.72
Oral health problem	0.56
Mechanical complication of device, implant, graft, catheter	0.56
Complications of transplant (kidney, liver, heart, lung)	0.53
Angioneurotic edema	0.43
Obstetric or pregnancy problem	0.42
Abnormal coagulation profile	0.42
Pulmonary embolism	0.35
Ventilator associated pneumonia, post-procedural aspiration pneumonia and other respiratory complication	0.34
Urinary complication	0.34
Disruption of wound	0.34
Iatrogenic stroke	0.31
Ophthalmological	0.30
Complication of tracheostomy, gastrostomy or colostomy	0.29
Infected amputation stump	0.26
Infection following infusion, injection, transfusion, or vaccination	0.26
Digestive system complication	0.24
Other complications of procedure or not specified	0.20
Rehabilitation care	0.17
Functional disturbance	0.16
Anaphylactic shock	0.15
Encounter for chemotherapy and immunotherapy	0.14
Sleep apnoea	0.13
Oedema	0.13

Venous disease including insufficiency and phlebitis	0.13
Accidental procedural laceration	0.11
Lymphatic disease including lymphedema	0.10
Complication of prosthetic joint (dislocation, fracture, etc)	0.09
Other amputation complication	0.09
Unspecified debility	0.06
Pneumothorax	0.06
Vaccination	0.06
Malnutrition	0.04
Surgical complication	0.04
Swell, mass or lump	0.04
Gangrene	0.04
Complication of medical care	0.04
Chemotherapy reaction	0.03
Transfusion reaction	0.02
Transient alteration of awareness	0.02
Nervous system complication	0.02
Inflammatory disease of breast	0.02
Flushing	0.02
Eating disorder	0.02
Other abnormal blood chemistry/blood finding	0.02
Postoperative shock	0.02
Serum reaction	0.02
Observation and evaluation for other suspected conditions	0.02
Anomalies of airways	0.01
Musculoskeletal anomalies	0.01
Pallor	0.01
Tuberculin test reaction	0.01
Nervousness	0.01
Complication of surgical and medical care	0.01
Adverse food reaction	0.01
Foreign body accidentally left during procedure	0.01
Air embolism	0.01
Complication of medical care	0.01
Infusion reaction	0.01
Fitting of vascular catheter	0.01
Encounter for drug monitoring	0.01

STROBE Statement: Checklist of items that should be included in reports of cohort studies

	Item No	Recommendation	Page No
**Title and abstract**	1	(*a*) Indicate the study's design with a commonly used term in the title or the abstract	1
		(*b*) Provide in the abstract an informative and balanced summary of what was done and what was found	2
**Introduction**			
Background/rationale	2	Explain the scientific background and rationale for the investigation being reported	3
Objectives	3	State specific objectives, including any prespecified hypotheses	3-4
**Methods**			
Study design	4	Present key elements of study design early in the paper	10-11
Setting	5	Describe the setting, locations, and relevant dates, including periods of recruitment, exposure, follow-up, and data collection	10-11
Participants	6	(*a*) Give the eligibility criteria, and the sources and methods of selection of participants. Describe methods of follow-up	10-11
		(*b*) For matched studies, give matching criteria and number of exposed and unexposed	NA
Variables	7	Clearly define all outcomes, exposures, predictors, potential confounders, and effect modifiers. Give diagnostic criteria, if applicable	10-11
Data sources/ measurement	8*	For each variable of interest, give sources of data and details of methods of assessment (measurement). Describe comparability of assessment methods if there is more than one group	10-11
Bias	9	Describe any efforts to address potential sources of bias	10-11
Study size	10	Explain how the study size was arrived at	10-11
Quantitative variables	11	Explain how quantitative variables were handled in the analyses. If applicable, describe which groupings were chosen and why	10-11
Statistical methods	12	(*a*) Describe all statistical methods, including those used to control for confounding	10-11
		(*b*) Describe any methods used to examine subgroups and interactions	10-11
		(*c*) Explain how missing data were addressed	10-11
		(*d*) If applicable, explain how loss to follow-up was addressed	10-11
		(*e*) Describe any sensitivity analyses	10-11
**Results**			
Participants	13*	(a) Report numbers of individuals at each stage of study—eg numbers potentially eligible, examined for eligibility, confirmed eligible, included in the study, completing follow-up, and analysed	5-6
		(b) Give reasons for non-participation at each stage	Figure 1
		(c) Consider use of a flow diagram	Figure 1
Descriptive data	14*	(a) Give characteristics of study participants (eg demographic, clinical, social) and information on exposures and potential confounders	
		(b) Indicate number of participants with missing data for each variable of interest	Figure 1
		(c) Summarise follow-up time (eg, average and total amount)	5-6
Outcome data	15*	Report numbers of outcome events or summary measures over time	5-6
Main results	16	(*a*) Give unadjusted estimates and, if applicable, confounder-adjusted estimates and their precision (eg, 95% confidence interval). Make clear which confounders were adjusted for and why they were included	5-6
		(*b*) Report category boundaries when continuous variables were categorized	5-6

		(*c*) If relevant, consider translating estimates of relative risk into absolute risk for a meaningful time period	5-6
Other analyses	17	Report other analyses done—eg analyses of subgroups and interactions, and sensitivity analyses	5-6
**Discussion**			
Key results	18	Summarise key results with reference to study objectives	7-9
Limitations	19	Discuss limitations of the study, taking into account sources of potential bias or imprecision. Discuss both direction and magnitude of any potential bias	7-9
Interpretation	20	Give a cautious overall interpretation of results considering objectives, limitations, multiplicity of analyses, results from similar studies, and other relevant evidence	7-9
Generalisability	21	Discuss the generalisability (external validity) of the study results	7-9
**Other information**			
Funding	22	Give the source of funding and the role of the funders for the present study and, if applicable, for the original study on which the present article is based	14

*Give information separately for exposed and unexposed groups.

Note: An Explanation and Elaboration article discusses each checklist item and gives methodological background and published examples of transparent reporting. The STROBE checklist is best used in conjunction with this article (freely available on the Web sites of PLoS Medicine at http://www.plosmedicine.org/, Annals of Internal Medicine at http://www.annals.org/, and Epidemiology at http://www.epidem.com/). Information on the STROBE Initiative is available at http://www.strobe-statement.org.
